# A Data-Driven Approach for Building the Profile of Water Storage Capacity of Soils

**DOI:** 10.3390/s23125599

**Published:** 2023-06-15

**Authors:** Jiang Zhou, Ciprian Briciu-Burghina, Fiona Regan, Muhammad Intizar Ali

**Affiliations:** 1 School of Electronic Engineering, Dublin City University, D09 DXA0 Dublin, Ireland; jiang.zhou@insight-centre.org; 2 DCU Water Institute, Dublin City University, D09 K20V Dublin, Ireland; ciprian.burghina2@mail.dcu.ie (C.B.-B.); fiona.regan@dcu.ie (F.R.); 3 School of Chemical Sciences, Dublin City University, D09 K20V Dublin, Ireland

**Keywords:** hydrology, LSTM, water storage capacity, soil moisture

## Abstract

The soil water storage capacity is critical for soil management as it drives crop production, soil carbon sequestration, and soil quality and health. It depends on soil textural class, depth, land-use and soil management practices; therefore, the complexity strongly limits its estimation on a large scale with conventional-process-based approaches. In this paper, a machine learning approach is proposed to build the profile of the soil water storage capacity. A neural network is designed to estimate the soil moisture from the meteorology data input. By taking the soil moisture as a proxy in the modelling, the training captures those impact factors of soil water storage capacity and their nonlinear interaction implicitly without knowing the underlying soil hydrologic processes. An internal vector of the proposed neural network assimilates the soil moisture response to meteorological conditions and is regulated as the profile of the soil water storage capacity. The proposed approach is data-driven. Since the low-cost soil moisture sensors have made soil moisture monitoring simple and the meteorology data are easy to obtain, the proposed approach enables a convenient way of estimating soil water storage capacity in a high sampling resolution and at a large scale. Moreover, an average root mean squared deviation at $0.0307\;m^{3}/m^{3}$ can be achieved in the soil moisture estimation; hence, the trained model can be deployed as an alternative to the expensive sensor networks for continuous soil moisture monitoring. The proposed approach innovatively represents the soil water storage capacity as a vector profile rather than a single value indicator. Compared with the single value indicator, which is common in hydrology, a multidimensional vector can encode more information and thus has a more powerful representation. This can be seen in the anomaly detection demonstrated in the paper, where subtle differences in soil water storage capacity among the sensor sites can be captured even though these sensors are installed on the same grassland. Another merit of vector representation is that advanced numeric methods can be applied to soil analysis. This paper demonstrates such an advantage by clustering sensor sites into groups with the unsupervised K-means clustering on the profile vectors which encapsulate soil characteristics and land properties of each sensor site implicitly.

## 1. Introduction

Soil moisture represents the water content of the soil, which is strongly affected by the storage and movement of water in the soil. Several indicators have been proposed to infer the ability of holding water in soil such as saturated water content and field capacity. However, these indicators are static measurements of the amount of water in the soil at a specific time. They do not take into account the variability in soil moisture and the changes in soil properties or climatic conditions over time. The same weaknesses are also shared in a soil water characteristic curve (SWCC), which represents a single snapshot of the soil’s water-holding capacity at a given point in time. Water storage capacity of soil, on the other hand, is not limited to a specific point in time. It describes the amount of water that a soil can hold under various moisture levels over a range of time periods. It takes soil dynamics into account as well as environmental factors, such as precipitation, evapotranspiration, etc.; thus, the modelling of water storage capacity becomes very complicated and difficult. For example, the space between soil particles can be filled with water as well as air, the physicochemical interactions between soil and water can alter the density of soil water, and the relationship between soil moisture and runoff responses can be nonlinear and is attributed to many factors such as topography, soil properties, vegetation, etc. [1]. Many methods have been proposed to model the water storage capacity of soil from various perspectives, such as pore geometry [2], soil physical properties [3], initial wetness conditions [4], soil texture and organic matter [5], hydrological soil properties [6], etc. However, it is impossible to take all impact factors explicitly into account in a model.

Recently, the data-driven approach, which infers soil information directly from the data without considering the underlying physical processes, has become popular. Following this trend, in this paper, a neural network approach is proposed to build a profile of soil water storage capacity, without knowing the principle of water conservation or the governing processes such as infiltration or evapotranspiration, etc., a priori, but learning them entirely from the data supplied. The proposed neural network is based on LSTM, a type of recurrent neural network capable of capturing highly nonlinear relationships and handling long-term dependencies in sequential data. The neural network takes the meteorology data as predictor variables and the in situ soil moisture as target variables. Seven months of in situ soil moisture data from 10 capacitance-based sensors deployed on 10 experimental sites, together with corresponding meteorology data, are collected to build the models. The cell state vectors in the built LSTM models are then extracted out as the profiles of the soil water storage capacity for the 10 sensor sites. Comparing to single value indicators, a multidimensional vector has the ability to encode the soil responses to various impact factors over time and thus is a more powerful representation. The profile vector encapsulates soil properties and dynamics implicitly, and thus provides a convenient tool for further soil analysis with numerical methods, which will be demonstrated in this paper for anomaly detection and categorization.

In contrast to [7], our algorithm keeps updating the same cell state vector of all cells during the entire training. In our approach, updating the internal cell state is regulated through the LSTM gates. The input gate regulates the increase in the storages, the forget gate regulates the depletion of the storages, and the output gate regulates the output of the storages. The training takes sequences of 30 h meteorology data and outputs soil moisture estimation for every input hour. The estimated soil moisture is compared to the in situ soil moisture, and the errors are backpropagated to update the neural network as well as the cell state vector. In this way, using soil moisture as a proxy, the cell state vector learns the moisture response to the various changes in soil in terms of water content and builds the profile of the water storage capacity of the soil. It can be seen from our experiments that, even for the same land type, the subtle difference in water storage capacity from different sensor sites can be captured with these profile vectors.

Our trained models can achieve an average root mean squared deviation (RMSD) of 10 sensor sites at $0.0307\;m^{3}/m^{3}$ in soil moisture estimation, which is less than $0.04\;m^{3}/m^{3}$, a general accuracy desire of soil moisture retrieval in many applications. Therefore, the trained models can also be deployed as an alternative to expensive sensor networks for continuous soil moisture monitoring. Sensor networks are usually deployed for real-time soil moisture monitoring; these networks, however, are limited to sparse monitoring locations and small coverage areas due to the cost, deployment, and network communication challenges. Using the proposed approach, a soil moisture network can be expanded further without physically installing a sensor on every monitoring location. Dense monitoring of a soil moisture network can also be achieved even after a sensor is removed from a monitoring location as long as a model, which only needs the meteorology inputs in deployment, is built for that particular location.

### 1.1. Soil Hydrology Modelling

Water storage and drainage in soil are essential steps in the hydrologic cycle. Nachabe et al. [8] introduced a model to estimate soil water storage capacity using observations of shallow water table fluctuations and soil moisture in shallow, sandy soil. However, the estimation requires the consideration of many impact factors explicitly such as encapsulated air, the capillary fringe, and soil texture heterogeneity. Sheikh et al. [3] introduced a simple two-layer soil water balance model to predict soil moisture, which utilizes daily meteorological records, soil physical properties, basic crop characteristics, and topographical data. The root mean squared error of predicted soil moisture content for their experimental locations ranged from 0.011 to 0.065 ${\mathrm{cm}}^{3}{\mathrm{cm}}^{-3}$. Alves et al. [2] suggested a model to predict the soil water characteristic curve based on pore-scale analysis and three-dimensional approximations of pore geometry using unit cells. The proposed model considers the effect of particle size and packing porosity on retention and provides reasonable results for drying SWCCs, offering a general approach that may be modified in the future.

In general, rainfall–runoff models are the standard tools used for investigating hydrological processes [9]. Matteo [6] reconstructed the SWCC with the Soil Water Characteristic software [5] to understand the infiltration processes in unsaturated soils. Song and Wang [4] conducted artificial rainfall–runoff experiments to investigate the nonlinear patterns of rainfall–runoff response. The study found that soil moisture data can provide valuable insights into the processes of runoff generation in hydrology. Singh et al. [1] also revealed that soil moisture responses are influenced by a combination of storm properties and landscape characteristics, which in turn affect the relationship between soil moisture and runoff during storms.

### 1.2. Long Short-Term Memory Modelling

Long short-term memory (LSTM) is an artificial neural network for sequence modelling [10,11,12]. Li et al. [13] built a data-driven model with LSTM for streamflow prediction on a 15-minute scale using precipitation as the only input. Compared to the process-driven gridded surface subsurface hydrologic analysis (GSSHA) model, the data-driven model is clearly more efficient and robust in terms of prediction and calibration. Li et al. [14] developed an attention-aware LSTM model for soil moisture and soil temperature prediction. They experimented with 1 day and 7 days flux tower data in a sequence for the soil moisture prediction and obtained the root mean squared errors of 10 experiment sites from 1.178 to 3.865 at the lead time of 1 day. O and Orth [15] trained an LSTM model to extrapolate daily soil moisture dynamics in space and in time, based on in situ data collected from more than 1000 stations across the globe. The daily meteorological time series and static features obtained from both reanalysis and remote sensing datasets were used as the inputs to the LSTM, and the adjusted in situ soil moisture measurements were used as the training targets. Fang and Shen [16] trained LSTM with sequences of climatic forcings and physiographic attributes, such as soil properties and land cover attributes, and targetedthe Soil Moisture Active Passive (SMAP) L3 passive radiometer product for near-real-time forecasts of SMAP-based soil moisture.

Kratzert et al. [7] pointed out that the internal cell states of LSTM can be interpreted as some kind of storage such as snow accumulation, soil water content, or groundwater storage. They trained a regional hydrological model using LSTM to investigate the potential of LSTM for simulating runoff from meteorological observations, and demonstrated that the evolution of a cell state in the LSTM matches the dynamics of the temperature as well as the understanding of snow accumulation and snow-melt. Lees et al. [17] further investigated what information the LSTM captures about the hydrological system and argued that LSTMs can be used to gain an estimate of intermediate stores of water. In their study, it was shown that the state cell vector of the LSTM reflects known hydrological concepts such as soil water storage and snow processes, which are important for discharge generation.

## 2. Materials and Methods

### 2.1. Experimental Field and Data Collection

**Figure 1:**
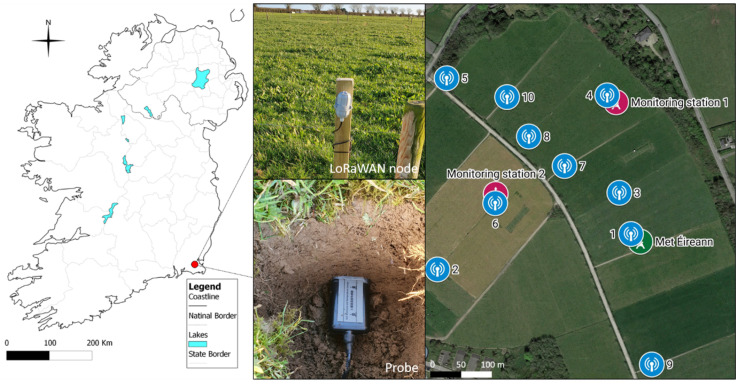
Sensor network deployment. (**Left**) Johnstown Castle site (red marker) and the map of Ireland; (**middle**) sensor installation example, showing the probe and the LoRaWAN node; (**right**) aerial site view showing the sensor locations (1–10), the meteorological station (Met Éireann), and the location of the LoRaWAN Gateway (monitoring station 1).

The soil moisture data were collected using in situ sensors deployed at a grassland site, Johnstown Castle, Wexford, Ireland (Figure 1). An LoRaWAN Outdoor Gateway (part number 102991154) and 4 × soil moisture and temperature sensors (part number 101990564) were procured from Mouser Electronic, Buckinghamshire, UK, with the remaining 6 units procured from DigiKey, Ireland (Thief River Falls, MN, USA). A 4.5 dBi LoRa antenna, 868 MHz, was procured from Paradar, London, UK, while the antenna extension cable was procured from Radionics, Dublin, Ireland. The meteorological data were obtained from a nearby Met Éireann station (Figure 1). The sensors were deployed at approximately 5 cm below the root line (Figure 1). All 10 sensor units used in this study were previously tested in the laboratory with both liquid and soil media. Unit-specific standardization equations in dielectric permittivity standards were developed for each node previously [18]. This was found to reduce the intersensor variability and provided robust estimates of volumetric soil moisture (θ) in soil samples with known θ, and when the sensors were tested against a TDR instrument, the two probes were found to be in good agreement throughout the tested range [18].

### 2.2. Long Short-Term Memory

**Figure 2:**
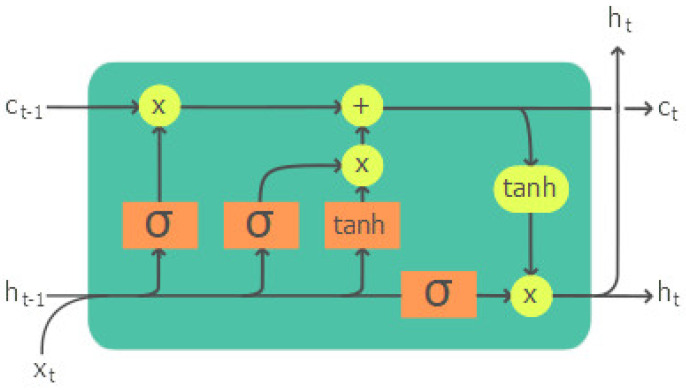
An illustration of a sample LSTM cell [19].

LSTM is a special kind of recurrent neural network (RNN). The core lies in its cell structure, as shown in Figure 2. There are two states in a cell, the hidden state *h* and the cell state *C*. It is the cell state *C* that alleviates the vanishing or exploding gradient problem. The cell state encodes long-term dependencies in a sequence and acts as the long-term memory. LSTM applies gates in its cell structure to regulate the information flowing through the sequence chain. There are three gates of LSTM, the forget gate, the input gate, and the output gate, in controlling the cell state to store or load information. The forget gate decides what information to dump from the cell state at time step *t*. The decision is made from the output of a sigmoid function δ, which takes the hidden state from previous time step $h_{t-1}$ and current input $x_{t}$, as shown in Equation (1), where *W* stands for the gate weight matrix and *b* is the bias.

(1) $$\begin{array}{c} f_{t}=\delta \left(W_{f}\cdot \left[h_{t-1},x_{t}\right]+b_{f}\right) \end{array}$$

The input gate decides what new information to store in the cell state. It is calculated as Equation (2), and a new vector $\tilde{C_{t}}$ is created from Equation (3) to contain all possible values that can be added to the cell state, where tanh stands for a hyperbolic tangent function. Then, the cell state can be updated with Equation (4).

(2) $$i_{t}=\delta \left(W_{i}\cdot \left[h_{t-1},x_{t}\right]+b_{i}\right)$$

(3) $$\tilde{C_{t}}=\tanh\left(W_{C}\cdot \left[h_{t-1},x_{t}\right]+b_{C}\right)$$

(4) $$C_{t}=f_{t}*C_{t-1}+i_{t}*\tilde{C_{t}}$$

Finally, the output gate decides which parts of the cell state to output in the next hidden state with Equations (5) and (6).

(5) $$O_{t}=\delta \left(W_{o}\cdot \left[h_{t-1},x_{t}\right]+b_{o}\right)$$

(6) $$h_{t}=O_{t}*\tanh\left(C_{t}\right)$$

**Figure 3:**
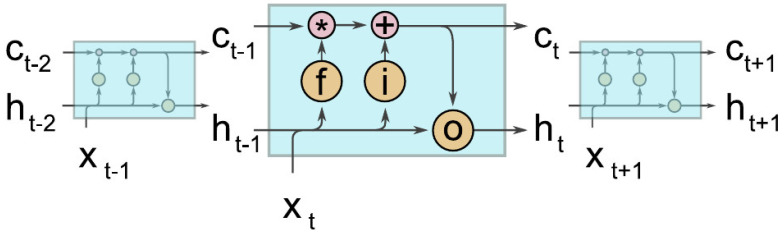
An illustration of a sample LSTM network [7].

An LSTM network is formed by assembling repeating LSTM cells in a chain, as shown in Figure 3. Given a sequence of input $\left[x_{1},x_{2},\ldots ,x_{t-1},x_{t},x_{t+1}\right]$ and initial states $h_{0}$ and $C_{0}$, a sequence of output $\left[h_{1},h_{2},\ldots ,h_{t-1},h_{t},h_{t+1}\right]$ can be computed.

### 2.3. Our Model

**Figure 4:**
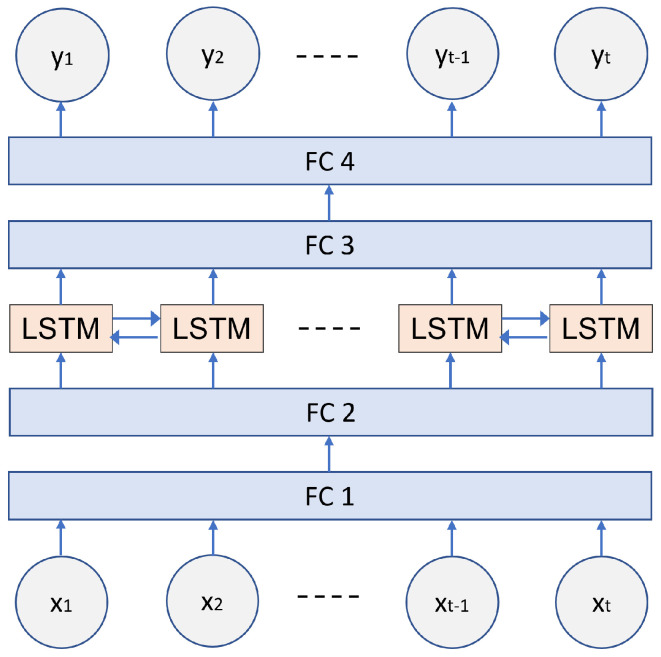
An illustration of our proposed model.

Given a sequence of meteorology data in a time span, a model is proposed, as depicted in Figure 4, to estimate the corresponding soil moisture response. The model is built upon single-layer bidirectional LSTM (bi-LSTM) which takes inputs from two directions, from left to right and from right to left. To account for the nonlinearity between the meteorology data and the soil moisture response, two fully connected layers, FC2 and FC3, are added before the input and after the output of the bi-LSTM, respectively, on every time step. Each of these two layers is followed by a sigmoid activation function. Note that the input dimension of FC3 is twice that of the output dimension of the bi-LSTM. This is because the output of the bi-LSTM on every time step contains vectors from two directions and the two output vectors are concatenated as the input to the layer FC3. The fully connected layer FC1 is the input layer of the model, which linearly maps a feature vector of the meteorology data to the size of the input dimension of layer FC2. The fully connected layer FC4 is the output layer of our model, which maps the dimension of the output vector from layer FC3 to size 1 linearly. Both FC1 and FC4 have no nonlinear functions followed.

The proposed model outputs a sequence of predicted soil moisture values. These values are compared to in situ soil moisture measurements which are used as the ground truth in a mean squared error (MSE) loss function during the model training. The training is optimized with the stochastic gradient descent method under L2 regularization. The initial states $h_{0}$ and $C_{0}$ in LSTM are generally set to zeros for each training sequence in every training epoch. However, our neural network is designed to learn the long-term mechanism of interaction between the meteorology data and soil moisture response implicitly, where the cell state *C* is modelled as the profile of the water storage capacity in the soil and it is nonlinearly affected by many factors, such as vegetation, soil properties, land surface topography, etc. Therefore, in the training, the cell state *C* starts from a vector with all zeros but keeps updating with every training sequence for all the training epochs, such that the cell state can be continuously regulated by many hydrological factors implicitly through the training data. Algorithm 1 shows how the training updates the cell state of our model.
**Algorithm 1** The update of the cell state.Initialize a vector $v=\vec{0}$**for** epoch = 1 to *N*
**do**      ▹ *N* is the total number of epochs **for** each pair (x,y) in *S*
**do**      ▹ *S* stands for all training sequences  Initialize $h_{0}=\vec{0}$  Initialize $C_{0}=v$  **for** t = 1 to *T*
**do**      ▹ *T* is the length of a sequence   calculate $f_{t}$ (Equation (1)), $i_{t}$ (Equation (2)), $\tilde{C_{t}}$ (Equation (3)),   update cell state $C_{t}$ (Equation (4))   calculate $O_{t}$ (Equation (5)), $h_{t}$ (Equation (6))  **end for**  Set $v=C_{t}$ **end for****end for**Output v as the final vector of the cell state

The trained model can be deployed for soil moisture estimation by supplying the meteorology data only. The cell state vector *C* can also be extracted out from the trained model and represent the profile of the water storage capacity of measured soil. Since our model is based on bi-LSTM, the profile vector is actually a concatenation of two cell state vectors from the bi-LSTM. One is from the bi-LSTM trained from left to right, and the other is from the bi-LSTM trained from right to left.

## 3. Results

### 3.1. Soil Moisture Estimation

We build our models for soil moisture estimation with about 3 months’ data from 13 December 2021 to 28 February 2022. A base model is trained first with some parameter searching, then models are built for each sensor site, respectively, by fine-tuning the base model. The 10 trained models are evaluated with 4 more months’ data, from 15 March 2022 to 30 June 2022, afterwards to test the performance consistency of the proposed method. Finally, another cycle of training, evaluation, and testing is completed with all available data from 13 December 2021 to 30 June 2022.

Both the meteorology data and the in situ soil moisture data are preprocessed with Z-score normalization. In this way, all features are centred around zero with a unit standard deviation, which would ease the learning during the model training. Our bi-LSTM model is trained with sequences of meteorology data as the input and sequences of in situ soil moisture data as the target output. To form a sequence of meteorology input, a start datetime $t_{1}$ is picked and the end datetime $t_{T}=t_{1}+T$ is then determined, where *T* is the length of the sequence in terms of hours. The hourly recorded meteorology data between $t_{1}$ and $t_{T}$ are segmented to form a sequence $\left[x_{1},\ldots ,x_{T}\right]$. Correspondingly, the soil moisture data between $t_{1}$ and $t_{T}$ are extracted to form the target sequence $\left[y_{1},\ldots ,y_{T}\right]$. Moving temporally along the meteorology data as a sliding window until *T* hours away from the end and using every meteorology record as a start of a sequence, we are able to build a dataset with sequences of meteorology data and sequences of in situ soil moisture data. For 10 sensor sites, 10 such datasets are constructed separately.

#### 3.1.1. Train a Base Model

To train a based model, 10% data are sampled from each dataset without overlapping each other and are pooled together to form the training set as well as the validation and testing sets. After removing a few corrupted sequences, the pooled dataset contains 805 sequences for training, 258 sequences in validation, and 368 sequences for testing.

The soil moisture response following rainfall events is unique for each location. This could confuse the learning during the base model training. However, all 10 sensors are installed on grassland. The in situ soil moisture readings from different sites should reveal some common responses of grassland to precipitation, condensation, and evaporation. The training, therefore, can still converge to a certain level, and some common characteristics of grassland would be encoded in the trained model.

We train the base model using 1000 epochs with 100 samples in each batch. Adam optimization [20] is used in training with a learning rate of 0.01, and L2 regularization is applied with weight decay 0.05. However, the dimension *D* of the hidden state *h* in our model must be determined before starting the training. This is also the size of the cell state *C*, which would have an effect on the representative power of the profile vector of the water storage capacity. A small dimension for the state vectors may lack the capability of capturing essential information, while a big dimension may lead to the curse of dimensionality during training and result in poor generalization to the model.

Therefore, a range of dimensions is evaluated. The length *T* of all sequences is set to 12 initially, which means that all sequences contain 12 h data. The base model is then trained and tested with hidden states in dimensions 8,16,32,64,128, and 256. We train 5 models for each dimension, and the average MSE in testing is reported in Table 1. As shown in the table, the dimension 128 gives the lowest testing MSE. We, therefore, set the dimension *D* of the hidden state and the cell state to 128 and fix it for the rest of the experiments in our evaluation.

The parameter *T* is also important for building the profile of the water storage capacity of the soil. It determines how many hours of weather changes show a delayed effect on soil moisture. Therefore a range of sequence lengths *T* is evaluated. Similar to the evaluation of the hidden state dimension, 5 models are trained and tested for each sequence length listed in Table 2. The average testing MSEs are reported. As shown in Table 2, a sequence length of 3,6,12,18,24,30 h are tested and the sequence length 30 gives the lowest MSE. This is expected, since the longer a sequence is, the more information is available for an estimation.

#### 3.1.2. Fine-Tune Models for Estimation

After the base model training, the dimension of the hidden state and cell state in our model is set to 128 and the input sequence length is fixed to 30. A model with the lowest MSE from the 5 models trained with D=128 and T=30 is selected as the base model. We then train models for each sensor site separately by fine-tuning the base model with the 3 months’ data from each sensor site, respectively. The training hyperparameters are the same as the ones used in the base model training, except that a learning rate of 0.001 is applied. Similar to the base model training, 5 models for each sensor site are trained and the average RMSD to the in situ soil moisture readings in testing is reported in Table 3. Calculating the mean value of RMSDs from all sensor sites, we obtain an RMSD of $0.0307\;m^{3}/m^{3}$ in soil moisture estimation. This is lower than RMSD $0.04\;m^{3}/m^{3}$, the accuracy requirements of soil moisture products from the satellites Soil Moisture and Ocean Salinity (SMOS) [21] and Soil Moisture Active Passive (SMAP) [22].

**Figure 5:**
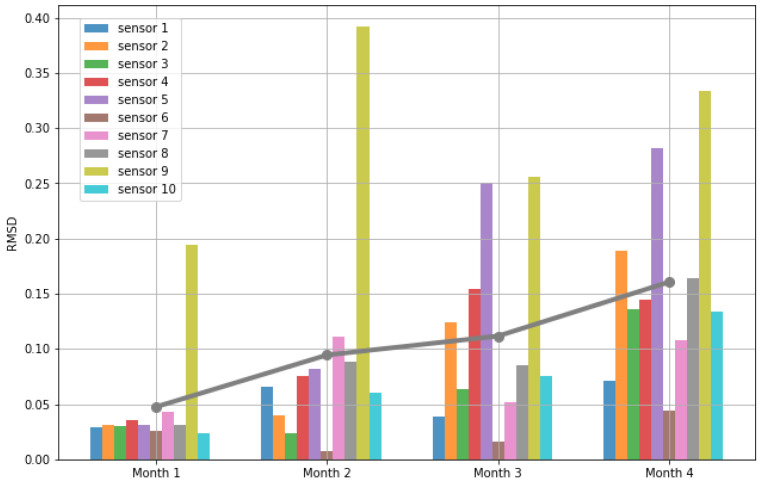
Model testing with 4 months’ data. The grey line indicates average RMSDs for each month.

The trained models are further evaluated with 4 more months’ data collected in the year 2022. Month 1 is from 16 March to 31 March, month 2 is from 1 April to 30 April, month 3 is from 1 May to 31 May, and month 4 is from 1 June to 30 June. Figure 5 plots the RMSDs of the 10 sensor site, and Table 4 lists the average RMSDs in each month, which are also plotted as the grey line in Figure 5. It can be seen in Figure 5 that the RMSD increases along with the months. Our models were trained with 2 months’ data collected in the winter time from 13 December 2021 to 30 January 2022. As the months move and enter different seasons, the weather and the soil moisture response change significantly; the information encapsulated in our trained models would, thus, gradually lose its representative power.

We also pool all the data available for another cycle of training, evaluation, and testing. Following a 80–20 split, the new models are trained with the 3 months of data from 13 December 2021 to 28 February 2022 plus data from 16 March 2022 to 24 April 2022, and are tested with data from 24 May 2022 to 30 June 2022. The average RMSD of the new trained models in testing is $0.145\;m^{3}/m^{3}$. This is close to the RMSD of the previous models evaluated in month 4 listed in Table 4. As shown in Table 4, the mild RMSD increase in months and suggests that the data between 16 March and 24 April from the spring season would only present a small amount of additional information to the previous 3 months’ data captured in the wintertime. The new trained models, therefore, would experience the same difficulty as the previous models in estimation with data captured in the summertime.

### 3.2. The Profile of Soil Water Storage Capacity

**Figure 6:**
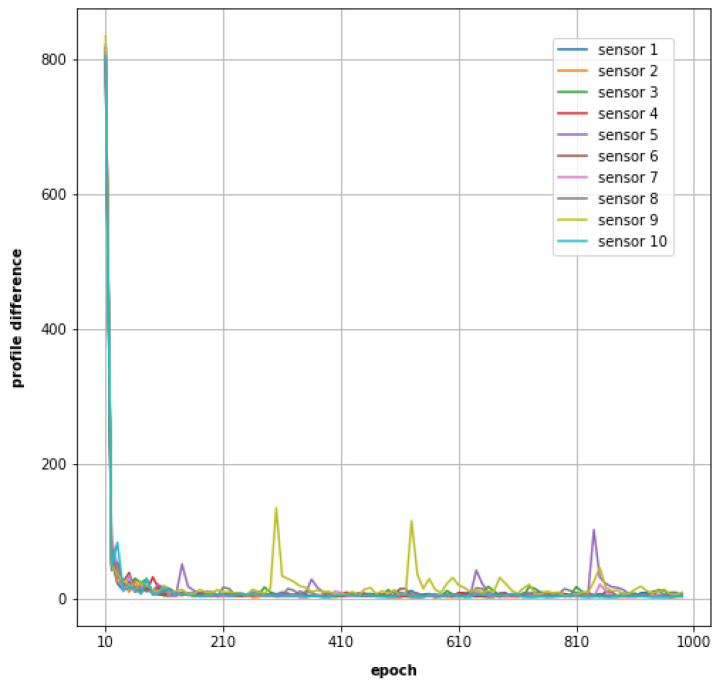
Profile vector differences along with training epochs.

The profile of the water storage capacity of the soil is modelled by the cell state vector of the LSTM. Since bi-LSTM is used in our neural network, the two 128 dimensional cell state vectors from the bi-LSTM are concatenated to form a single profile vector with a dimension of 256. For 10 sensor sites, 10 such profile vectors can be produced. Figure 6 depicts the evolution of profile vectors during the model training. The value of every point on a plot line is a Euclidean distance between two profile vectors which are from the same model training but 10 epochs apart. Along with the training, it can be seen that for all the sensor sites, the profile differences become small and the vectors become stable, even though there is still a bit of oscillation near the end of each model training. The cell state vector in our model is trained without explicit knowledge of the hydrological processes but is continuously regulated by the data with hydrological information embedded. When the cell state vector becomes stable in the training, it is deemed that the behaviour of the hydrological system has been deduced from the data and captured in the cell state vector. Our method estimates soil moisture, an indicator of the quantity of water existing in soil, from readily observed meteorology data; we, therefore, believe that the cell state vector of the model has learned the water storage capacity of the soil from the training and the vector can be used to characterize the soil in the numerical analysis, as demonstrated in the rest of this section.

#### 3.2.1. Anomaly Detection

**Figure 7:**
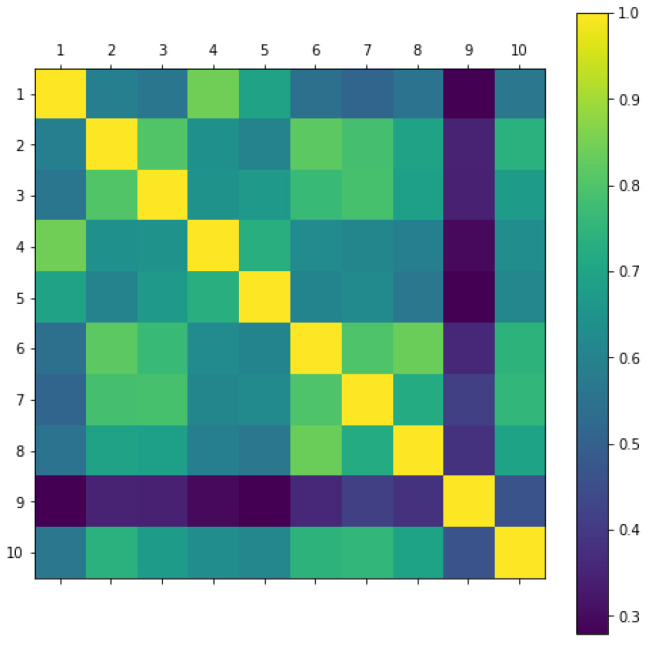
Pairwise Pearson correlation coefficients of 10 sensor sites.

In the experiments, five models are trained for each sensor site, which yields five profile vectors. We calculate the mean value of the five profile vectors along each dimension and use the averaged vector as the final profile vector for a sensor site. Once the profile vectors are computed for all sensor sites, the pairwise Pearson correlation coefficients of the 10 profile vectors are calculated, as shown in Figure 7. From the matrix shown in the figure, sensor site 9 can be easily detected as an anomaly since its profile vector has relatively low Pearson’s r values to all the other vectors. This is because sensor 9 had been deployed at a location which tends to be waterlogged during the rainy season, with poor drainage characteristics. However, as has been demonstrated, this anomaly detection is driven entirely by the sensor readings without knowing the real physical setup of each sensor site.

#### 3.2.2. Profile Validation and Categorization

**Figure 8:**
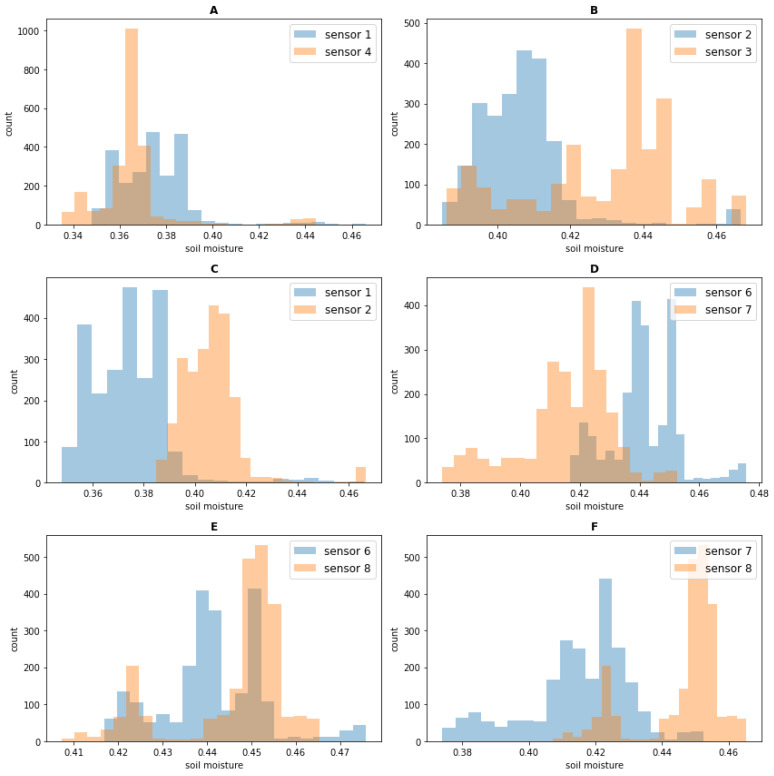
Soil moisture histograms of sensor sites. (**A**–**F**) represent 6 different sensors pair groups. Dark brown color in the histograms represents the overlapped region.

It can be seen that all the correlation coefficients in Figure 7 are positive, which can lead to a conclusion that the land type for all sensor sites is the same and, indeed, this is the case, as all our sensors are installed on the same grassland. Moreover, some patterns can be perceived from Figure 7, which suggests that some subtle soil difference from the 10 sensor sites could be discerned, even though all sites are from the same grassland. As shown in Figure 7, profiles from sensor sites 1 and 4 reach a high correlation. This tallies with plot A in Figure 8. In Figure 8, the histograms of the 2 months’ in situ soil moisture data used in training for some sensor sites are plotted. As plot A shows, the soil moisture histogram of sensor site 1 has a large overlapped region with the soil moisture histogram of sensor site 4, which implies that sensor site 1 has a similar profile of soil water storage capacity to sensor site 4. The same observation can also be found between sensor sites 2 and 3, where the high correlation between these two sites shown in Figure 7 tallies with the large overlapped region in plot B of Figure 8. In plot C of Figure 8, there is only a small overlapped region between sensor site 1 and sensor site 2, which implies that the profiles of soil water storage capacity of both sensor sites are different. This matches the low correlation between sensor sites 2 and 3 in Figure 7. Another interesting example can be given with sensor sites 6, 7, and 8. Sensor site 6 correlates well with both sensor sites 7 and 8, as shown in Figure 7; however, it can be seen that the overlapped region between sensor sites 6 and 8 in plot E of Figure 8 is larger than the overlapped region between sensor sites 6 and 7 in plot D. This is also reflected in the correlation matrix, where the square between sensor sites 6 and 8 in Figure 7 is a bit brighter than the square between sensor sites 6 and 7. Meanwhile, the dark-coloured square between sensor sites 7 and 8 reflects a relatively low correlation between the two sensor sites, and this can also be seen in plot F of Figure 8, where a small overlapped region occurs. Therefore, we can see that the profile vector is a representation of the soil water storage capacity and can reveal the subtle soil differences among many sensor sites.

Sensor sites could be grouped by computing overlapped regions between a pair of soil moisture histograms, as shown in Figure 8. However, when the number of sensor sites grows to hundreds, such a method would become intractable. In contrast, by taking the advantage of the vector representation, advanced machine learning algorithms can be applied for sensor site categorization. We demonstrate such an advantage by clustering sensor sites into groups using the unsupervised K-means clustering algorithm [23].

**Figure 9:**
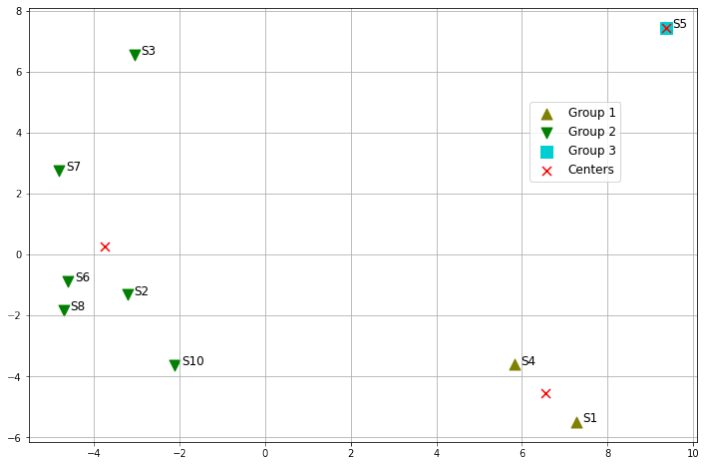
Categorization of 9 sensor sites. S stands for sensor site. S9 is excluded as an outlier.

Only nine sensor sites are used in this experiment since sensor site 9 is considered an outlier. Based on the correlation matrix in Figure 7, the number of groups is empirically set to three in the K-means algorithm. The categorization results are visualized in Figure 9 with the help of dimensionality reduction using principal component analysis (PCA). Table 5 summarizes the groups and lists the Euclidean distances to the group centres for each sensor site.

As shown in Figure 9, sensor sites 1 and 4 are in the same group. Sensor site 5 is not close to any other sensor sites so it becomes a group by itself. This can also be seen in Figure 7, where most squares in column 5 are dimmed. All the other sensor sites are categorized into group 1 in the figure. However, sensor site 7 seems closer to sensor site 3 in Figure 9, but in Figure 7 the square between sensor site 2 and 3 shows a higher correlation. This is because all the points in Figure 9 come from the PCA mapping to a 2D space. The values on the x-axis come from the first eigenvector mapping while the values on the y-axis come from the second eigenvector mapping. Therefore, the x-axis is a bit more important than the y-axis in Figure 9. When comparing sensor site 7 and sensor site 2, sensor site 2 is closer to sensor site 3 in terms of the x-axis. When computing the Euclidean distances with original 256 dimensional profile vectors, the distance between sensor sites 2 and 3 is 11.95, while the distance between sensor sites 3 and 7 is 12.15.

Another benefit of the K-means clustering is that now each group can be represented by a centroid vector, which is marked as a red cross in Figure 9. These centroid vectors can be regarded as metadata and be further used in other analyses.

## 4. Discussion

Data augmentation was experimented with in our model training. We first added a small amount of Gaussian noise which has 0 mean and 0.05 variance to the meteorology data, then randomly cut off a short sequence which is less than half of the training sequence length from either the front or the back of the sequence. Zero padding was then applied to pad the cut sequence to its original length. With this augmentation, the size of the training sets was doubled. However, the experiments did not show significant improvement in accuracy with the augmented dataset but the training time was extended. Therefore, we did not deploy this augmented dataset in our experiments reported.

Figure 5 in Section 3.1.2 shows an increase of RMSDs over time. This suggests that there is an effective period for the profile computed with our method. Beyond this period, the profile of the water storage capacity of soil built in the model would drift away from the true capacity since the soil itself could be changing significantly. As shown in the experiments, the extracted profile can be effective and give promising performance within about 3 months in the same season. Over 3 months in a different season, a remeasuring and a recomputation would be expected.

## 5. Conclusions

In this paper, we presented a machine learning approach for soil moisture estimation and building the profile of the water storage capacity of the soil. We used data collected from an existing low-cost soil moisture network and combined it with the meteorological data to train a neural network based on LSTM, and the experiments showed very promising results in soil moisture estimation. The approach can be deployed as an alternative to expensive sensor networks for continuous soil moisture monitoring. Using the proposed approach, dense monitoring can be achieved and current soil moisture networks can be expanded. The profile of the soil water storage capacity is derived from data without explicit knowledge of the hydrological processes. The profile vector encapsulates soil properties and land properties implicitly and provides a convenient tool for soil analysis. By taking advantage of the vector representation, we performed anomaly detection and categorization on the profiles of sensor sites from a low-cost soil moisture network.

The proposed model was built upon LSTM. It can be easily extended to a sequence-to-sequence model where the decoder outputs a sequence of future soil moisture without meteorology observation. Moreover, the attention mechanism can be applied with the sequence-to-sequence model such that the relationship between many impact factors and the profile of the soil water storage capacity can be established through the attention weights. This would also greatly help us understand how different hydrological processes work on soil moisture.

## Figures and Tables

**Table 1:** A base model with T = 12 and varied dimension.

Dimension	8	16	32	64	128	256
MSE	2.3657	2.3460	2.1953	2.2952	2.1719	2.2402

**Table 2:** A base model with D = 128 and varied sequence length.

Sequence Length	3	6	12	18	24	30
MSE	2.1343	2.1656	2.1641	2.1808	2.0807	2.0070

**Table 3:** RMSDs of 10 sensor sites.

Sensor	1	2	3	4	5	6	7	8	9	10
RMSD	0.0116	0.0171	0.0302	0.0305	0.0332	0.0220	0.0085	0.0094	0.1377	0.0064

**Table 4:** Average RMSDs of 10 sensor sites in an additional 4 months.

Month	1	2	3	4
RMSD	0.0475	0.0945	0.1116	0.1606

**Table 5:** Distances to each group centre and categorization results.

Sensor	1	2	3	4	5	6	7	8	10
To center 1	4.57	12.45	15.62	4.57	14.41	13.51	15.11	14.82	12.26
To center 2	13.39	6.06	9.52	11.89	15.61	5.56	7.41	8.52	7.59
To center 3	15.50	17.15	16.84	14.72	0	17.19	17.25	18.40	17.23
Group	1	2	2	1	3	2	2	2	2

## Data Availability

Data used in this paper were collected as part of a national initiative (Terrain-AI http://www.terrainai.com, last accessed on 1 June 2023) to build a data monitoring, analytics, and visualization platform to support climate action decision making. This dataset will be made available on the Terrain-AI platform.
